# Involvement of metabolic components, volatile compounds, PR proteins, and mechanical strengthening in multilayer protection of cucumber plants against *Rhizoctonia solani* activated by *Trichoderma atroviride* TRS25

**DOI:** 10.1007/s00709-017-1157-1

**Published:** 2017-09-06

**Authors:** Justyna Nawrocka, U. Małolepsza, K. Szymczak, M. Szczech

**Affiliations:** 1 Department of Plant Physiology and Biochemistry, Faculty of Biology and Environmental Protection, University of Lodz, Banacha 12/16, 90-237 Lodz, Poland; 2 Institute of General Food Chemistry, Lodz University of Technology, Stefanowskiego 4/10, 90-237 Lodz, Poland; 3 Research Institute of Horticulture, Konstytucji 3 Maja 1/3, 96-100 Skierniewice, Poland

**Keywords:** Callose, Genes, Lignin, *Rhizoctonia solani*, *Trichoderma*, Volatile compounds

## Abstract

In the present study, the spread of *Rhizoctonia solani*-induced disease was limited when cucumber plants were pretreated with *Trichoderma atroviride* TRS25. The systemic disease suppression was related to TRS25-induced resistance (TISR) induction with simultaneous plant growth promotion. Protection of cucumber was related to enhanced activity of defense enzymes, e.g., guaiacol peroxidase (GPX), syringaldazine peroxidase (SPX), phenylalanine ammonia lyase (PAL), and polyphenol oxidase (PPO) as well as phenolic (PC) concentration increases in the conditions of hydrogen peroxide (H_2_O_2_) accumulation, resulting in thiobarbituric acid reactive substance (TBARS) decrease. Moreover, the obtained results indicated that TISR might depend on accumulation of salicylic acid derivatives, that is methyl salicylate (MeSA), ethylhexyl salicylate (EHS), salicylic acid glucosylated conjugates (SAGC), and β-cyclocitral as well as volatile organic compounds (VOC) such as Z-3-hexanal, Z-3-hexenol, and E-2-hexenal. The results point to important, not previously documented, roles of these VOC in TISR signaling with up-regulation of *PR1* and *PR5* gene characteristic of systemic acquired resistance (SAR) and of *PR4* gene, marker of induced systemic resistance (ISR). The study established that TRS25 enhanced deposition of callose and lignin in specialized plant cells, which protected vascular system in cucumber shoots and roots as well as assimilation cells and dermal tissues in shoots and leaves. These compounds protected cucumber organs against *R. solani* influence and made them more flexible and resilient, which contributed to better nutrition and hydration of plants. The growth promotion coupled with systemic mobilization of biochemical and mechanical strengthening might be involved in multilayer protection of cucumber against *R. solani* activated by TRS25.

## Introduction

*Rhizoctonia solani* is one of the most destructive soil-borne necrotrophs. The pathogen induces damping-off, blight, rot of roots, and shoots in a variety of crop plants including cucumber (Singh et al. 2002; Bartz et al. 2010; Saberi et al. 2013; Yousef et al. 2013). Management of the disease caused by *R. solani* is difficult because of great variability in the pathogen population and long-term survival in soil (Bartz et al. 2010; Taheri and Tarighi 2012). Chemical fungicides, mainly methyl bromide together with different cultural practices such as crop rotation, and methods that minimize contact of the plant with *R. solani* are not sufficiently effective (Melo and Faull 2000; Montealegre et al. 2003). Integrated protection against the pathogen includes biological control as an important alternative or component of the disease management (Montealegre et al. 2003; Yousef et al. 2013).

Fungal species of the genus *Trichoderma*, being prevalent in soil, have been extensively used as biological control agents (BCA) against a wide range of plant pathogens (Harman et al. 2004; Alfano et al. 2007; Hermosa et al. 2012; Zhang et al. 2016). *Trichoderma* employ several modes of action contributing to their biocontrol activity and these modes vary depending on the strain and environment. Some strains act directly against pathogens via mycoparasitism, competition, and antibiosis (Elad 2000; Melo and Faull 2000; Vinale et al. 2008; Monfil and Casas-Flores 2014; Vos et al. 2015); others promote plant growth (Harman et al. 2004; Aly and Manal 2009; Christopher et al. 2010; Yadav et al. 2011) or induce natural plant protection at the site of infection as well as at distance to the pathogen resulting in alteration of the plant systemic resistance (Harman et al. 2004; Singh et al. 2010; Hermosa et al. 2012; Yousef et al. 2013; López-Bucio et al. 2015; Vos et al. 2015; Zhang et al. 2016). Simultaneous cucumber growth promotion and up-regulation of systemic resistance against pathogens are observed less frequently and still need elucidation (Shoresh et al. 2005; Harman et al. 2012; Mathys et al. 2012).

In the previous studies, *Trichoderma* strains were listed among the effective mycoparasitic and antagonistic BCA of *R. solani* (Khara and Hadwan 1990; Harman et al. 2004; Yousef et al. 2013). Further analyses suggested that *Trichoderma*-induced resistance (TISR), rather than a direct antifungal influence against *R. solani* protected plants against this pathogen (Yousef et al. 2013). Since the necessity to investigate multifunctional *Trichoderma* strains simultaneously promoting plant growth, inhibiting pathogens, and enhancing plant protective barriers is emphasized (Shoresh et al. 2005; Harman et al. 2012; López-Bucio et al. 2015), the primary aim of the present study was to investigate the basis of the effective protection of cucumber plants against *R. solani* induced by newly identified *Trichoderma atroviride* TRS25 strain. Biochemical, molecular, and structural responses were studied as symptoms of TRS25-induced resistance (TISR). Starting from the biochemical protection, generation of hydrogen peroxide (H_2_O_2_) which may act as an antimicrobial agent, signaling molecule, and substrate for defense enzymes (Małolepsza and Różalska 2005; Nanda et al. 2010) was analyzed. Simultaneous studies focused on determination of the activities of antioxidant enzymes, including ascorbate (APX), guaiacol (GPX), and syringaldazine peroxidase (SPX) which maintain the content of reactive oxygen species (ROS) at a safe level and participate in mechanical strengthening of plant tissues (Nikraftar et al. 2013). Moreover, the studies aimed with phenylalanine ammonia lyase (PAL) and polyphenol oxidase (PPO) involved in the synthesis of antioxidant and antimicrobial active phenolic compounds (PC). Among PC, *orto*dihydroxyphenolics (*o*DP), phenylpropanoids (PP), favonoids (FL), and salicylic acid (SA) were studied as compounds which may contribute to resistance induction, direct suppression of pathogens, and to creation of structural barriers which prevent progress of the disease (Yedida et al. 2003; Park et al. 2007; Singh et al. 2011; Oliveira et al. 2016). To assess membrane stability, thiobarbituric acid reactive substance (TBARS) concentration was analyzed. Particular attention was paid to volatile compounds (VOCs) including methyl salicylate (MeSA), ethylhexyl salicylate (EHS), and β-cyclocitral as well as unsaturated fatty acids, that is, linolenic and linoleic acid derivatives, including Z-3-hexanal, Z-3-hexenol, and E-2-hexenal not studied extensively as compounds which together with H_2_O_2_ may be involved in resistance signaling resulting in enhanced expression of defense genes of pathogenesis-related (PR) proteins (Mathys et al. 2012; Brotman et al. 2013; Dudareva et al. 2013; Gao et al. 2014; Lv et al. 2015; De Palma et al. 2016; Adrian et al. 2017). Except of MeSA, these compounds were not considered among metabolic components playing the role of signaling molecules in plants in response to *Trichoderma* influence. To characterize the TISR induced by the detected VOC, studies of gene transcript expression were performed for *PR1* (acidic proteins) and *PR5* (thaumatin-like proteins) marker genes of the systemic acquired resistance (SAR) and for *PR4* (chitinase) and *PR12* (defensin) gene characteristic of induced systemic resistance (ISR). The tested genes are known as allowing to determine kind of resistance, basically, JA- and SA-dependent or both (Alfano et al. 2007; Salas-Marina et al. 2011; Hermosa et al. 2012; Perazzolli et al. 2012; Martinez-Medina et al. 2013; Nawrocka and Małolepsza 2013; Vos et al. 2015), which might be induced by the tested *Trichoderma*. Finally, we particularly focused on the deposition and location of stabilizing components such as callose and phenolic polymer, lignin important for plant mechanical strengthening. The structural barriers in plant cells may be related to crosslinking of proteins and deposition of the mentioned compounds to separate and protect susceptible tissues, to limit spread of the pathogen, or to delay the infection process until other defensive mechanisms become active (Solanki et al. 2011; Nikraftar et al. 2013; Rao et al. 2015). A common model of callose synthesis favors a deposition of this glucan in the paramural space between a cell wall and a plasma membrane in the form of papillae while lignin usually builds secondary walls of root vessel and dermal tissue cells in the area of pathogen influence (Chowdhury et al. 2014; Rao et al. 2015; Schneider et al. 2016). Since enhancement of structural barriers was mainly detected and analyzed in the tissues directly attacked by pathogens, we focused on systemic location of callose and lignin in the entire plant and their role not only in the protection against *R. solani* but also in cucumber growth promotion. Identification of the basis of TRS25-induced cucumber resistance including metabolic components, VOC, PR proteins, and mechanical strengthening with simultaneous plant growth promotion would aid to elucidate the complicated network activated by this strain effectively protecting cucumber plants against *R. solani*.

## Material and methods

### Fungal strains and plant material preparation

*T. atroviride* TRS25 was collected from a growing medium for mushroom production. TRS25 was confirmed as not pathogenic for the cultivated mushroom. The isolate was identified morphologically using an interactive key (http://nt.ars-grin.gov/taxadescriptions/keys/TrichodermaIndex.cfm), supported by identification keys provided by Gams and Bissett (1998) and Samuels et al. (2002). The identification was followed by molecular classification of the isolate performed by Oskiera et al. (2015). The microorganism sequences have been deposited at the NCBI GenBank (http://www.ncbi.nlm.nih.gov) with accession numbers: ITS KJ786731 and tef1α KJ786812.

Complementary tests examining antagonistic or mycoparasitic properties of TRS25 isolate against various pathogens including *R. solani* showed moderate ability of this strain to colonize, overgrow, or parasitize fungal sclerotia (Szczech et al. 2014). TRS25 isolate used to treat cucumber plants was grown on Malt Extract Agar media (Fluka) in petri plates for 10 days at 25 °C. Every 24 h, the cultures were exposed to light for 20 min to activate fungus sporulation. To obtain inoculum of TRS25, the spores of the fungus were washed off the surface of 10-day old cultures with 10 ml of 0.85% NaCl solution. *R. solani* Kühn MUCL47938 strain used in the experiment is a well-characterized, standard phytopathogen of cucumber plants. The fungus was grown for 7 days in 9-cm diameter petri plates on potato dextrose agar (PDA), in the dark, at the constant temperature of 25 °C. After incubation, mycelia mats of five plates were homogenized in 0.5 l of deionized water and used to inoculate cucumber plants.

Cucumber (*Cucumis sativus* L.) plants cv. Iwa F^1^, susceptible to *R. solani*, were used in the experiment. The plants were cultivated in the media consisting of podsolic soil and vermiculite 1:1 (*v*:*v*). To investigate the potential of TRS25 to induce resistance in cucumber plants, the growing medium was supplemented with aliquots of the spore suspension to obtain 10^6^ spore density per 1 g of the medium. The medium was thoroughly mixed and distributed to 0.5-dm^3^ plastic pots. Medium without TRS25 was used as control. Approximately 500 g of the medium with 60% water holding capacity adjusted with tap water was used in each pot and sown with one seed of cucumber per pot. Cucumber plants grew in a chamber (Sanyo, model MLR-351 H with 15 fluorescent lamps type FL 40SSW/37), under 14-h photoperiod with a 20,000-lx light intensity at 25/20 °C, day/night temperature cycle, and 80% relative humidity. The plants were watered daily with tap water. Seven-day-old cucumber seedlings were fertilized with Polyfeed (POLY-FEED VITA 12-10-34+2 HAIFA, Poland). Three weeks after sowing half of the control and TRS25, pretreated plants were inoculated with 5 ml of the *R. solani* mycelia homogenate near the cucumber stem base according to the method of this pathogen application presented by Pannecoucque and Höfte (2009). All plants were then maintained in the growth chamber for 7 days. Four experimental groups of plants were tested: (I) control plants—nontreated with TRS25 and uninoculated with *R. solani*, (II) Rs plants—nontreated with TRS25 and inoculated with *R. solani*, (III) TRS25 plants*—*pretreated with TRS25, uninoculated with *R. solani*, and (IV) TRS25 + Rs plants*—*TRS25 pretreated, challenged with *R. solani*. Twelve pots were prepared for each treatment. The experiment was prepared four times under the same conditions. Twenty-eight-day old cucumber plants were cut at the stem base. Their fresh weight (FW) was determined, and the third leaf of each plant was cut off for biochemical assays. In each experiment, the samples for biochemical analyses were prepared in triplicate. The irregular lesions and rot symptoms were observed on the roots of 5-week old Rs plants. Disease development on the root surfaces was evaluated using a Motic Images Plus 2.0^ML^ program (Motic China Group, Asia) according to the manufacturer’s instruction. Then, the roots were dried at 60 °C for 24 h and their dry weight (DW) was measured.

### Hydrogen peroxide (H_2_O_2_), thiobarbituric acid reactive substance, and protein contents

To measure H_2_O_2_ content, the compound was extracted from 500-mg samples from three fresh leaves according to the modified method of Capaldi and Taylor (1983), previously described by Małolepsza and Różalska (2005). The content was calculated based on the standard curve of H_2_O_2_ and expressed in μmol per g of FW.

As an index of lipid peroxidation, the TBARS content was determined according to the method of Yagi (1976) with modifications, as described by Nawrocka et al. (2011). TBARS content was estimated by referring to a standard 1,1,3,3-tetraethoxypropane (Sigma-Aldrich) and expressed in nmol per g of FW. The supernatants prepared for TBARS determination were also used to study protein content according to the method of Bradford (1976) with a standard curve prepared using bovine serum albumin (Sigma-Aldrich).

### Enzymatic activity

To measure ascorbate, guaiacol, and syringaldazine peroxidase (APX, EC.1.11.1.11; GPX, EC 1.11.1.7 and SPX, EC 1.11.1.7, respectively) activities, 250-mg samples from three fresh leaves were ground in an ice-cold mortar with 50-mM sodium phosphate buffer (1:5; *w*:*v*; pH 7.0) containing 1-mM EDTA, 1-mM sodium ascorbate, and 0.5-M NaCl and centrifuged (15,000 ×*g*, 15 min). APX activity was assayed using the modified method of Nakano and Asada (1981) following the oxidation of ascorbate to dehydroascorbate, which was determined at 265 nm. The enzyme activity was calculated based on absorbance coefficient *ε* = 13.7 mM^−1^ cm^−1^ and expressed in units (1 U = 1 μmol ascorbate oxidized min^−1^ mg^−1^ protein). GPX activity was estimated by measuring an increase in absorbance of tetraguaiacol (*ε* = 26.6 mM^−1^ cm^−1^), a colored product of guaiacol oxidation, at 470 nm for 4 min at 30 °C (Maehly and Chance 1954; Nawrocka et al. 2011). The enzyme activity was expressed in units (1 U = 1 mmol of tetraguaiacol formed min^−1^ mg^−1^ protein). SPX activity was assayed by the method of Imberty et al. (1985) following oxidation of syringaldazine (*ε* = 27 mM^−1^ cm^−1^). The absorbance increase was measured at 530 nm and the enzyme activity was expressed in units (1 U = 1 μmol of syringaldazine oxidized min^−1^ mg^−1^ protein).

To determine phenylalanine ammonia lyase (PAL; EC 4.3.1.24) activity, 500-mg samples from three fresh leaves were ground in an ice-cold mortar with 0.5-M Tris-HCl buffer (1:10; *w*:*v*; pH 8.8), containing 0.8-mM β-mercaptoethanol and 1% polyvinylpolypyrrolidone. PAL was determined according to Zucker (1965) by the production of trans-cinnamic acid from l-phenylalanine during 1 h at 37 °C. The enzyme activity was expressed in units (1 U = 1 μmol of trans-cinnamic acid formed min^−1^ mg^−1^ protein). To measure polyphenol oxidase (PPO; EC 1.10.3.1) activity, 500-mg samples from three fresh leaves were ground in an ice-cold mortar with 50-mM sodium phosphate buffer (1:5; *w*:*v*; pH 7.0) containing 1-M NaCl, stirred for 30 min at room temperature and centrifuged (20,000 ×*g*, 20 min). PPO activity was determined according to the method of Chang et al. (2000) by measuring the increase in absorbance at 420 nm following the oxidation of catechol. One unit of PPO activity was calculated based on absorbance coefficient *ε* = 2.72 mM^−1^ cm^−1^ and expressed in units (1 U = 1 nmol catechol oxidized min^−1^ mg^−1^ protein).

### Phenolic compound content

To determine PC, *o*DP, PP, and flavonoid (FL) contents 500-mg samples from three frozen leaves were homogenized with 80% MeOH (1:10; *w*:*v*) and centrifuged (20,000 ×*g*, 20 min). PC content was determined by the method of Singleton and Rossi (1965) and *o*DP were determined with Johnson and Shaal (1957) method, as described previously by Nawrocka et al. (2011). PC and *o*DP contents were calculated using a standard curve prepared for chlorogenic acid (Sigma-Aldrich) and expressed in mg per g FW. PP content was measured in the crude extract obtained by mixing the supernatant with 0.1% HCl in 95% ethanol and 2% HCl, 1:18 (*v*:*v*) according to Glories (1978). The absorbance at 320 nm was measured. PP content was calculated on the basis of a standard curve of the caffeic acid (Sigma-Aldrich) and expressed in mg per g of FW. FL content was estimated using the modified method of Chang et al. (2002). The reaction mixture containing methanolic extract, 10% AlCl_3_ × 6H_2_O, and 1-M CH_3_COONa was incubated for 30 min at room temperature. The absorbance was measured at 234 nm. FL content was expressed in mg of the standard quercetin (5 mg/100 ml) (Sigma-Aldrich) per g of FW.

### Salicylic acid and salicylic acid glucosylated conjugate contents

SA was extracted according to the modified protocol of Molina et al. (2002). One-gram samples from frozen leaves were extracted three times in 80, 90, and then 100% (1:10; *v*/*v*) MeOH. After centrifugation (15 min, 20,000 ×*g*), all the supernatants were combined and evaporated to dryness under vacuum at 65 °C. The residue was redissolved in water at 80 °C and centrifuged (15 min, 20,000 ×*g*). Then SA was extracted three times into three volumes of cyclopentane:ethylacetate:2-propanol (50:50:1, *v*/*v*/*v*). The organic extracts were dried under vacuum and resuspended in 70% MeOH containing 0.5% fumaric acid (FA). To release SA from SAGC, the aqueous phase was acidified with HCl to pH 1.5–2.0 and boiled for 1.5 h at 80 °C. The released SA was extracted with the organic mixture as described above. HPLC system (DIONEX, Sunnyvale, CA, USA) was used for analysis. Separation of SA took place over an RP column (aQ Hypersil GOLD, 250 mm × 4.6 mm, 5 μm) joined with a guard column (GOLD aQ Drop-In guards, 10 mm × 4 mm, 5 μm) at 40 °C, by use of a binary solvent system consisting of (A) water and (B) methanol with 0.5% FA with a flow rate of 1.5 ml/min. The elution profile was as follows: 0–2 min, 40% B; 2–10 min, 40–60% B; 10–12 min, 60% B; 12–13 min, 60–40% B; and 13–15 min, 40% B. Chromatograms were obtained by fluorescence evaluation (excitation 301 nm, emission 412 nm). Quantification was based on the calibration curves for the adequate SA standards (Sigma-Aldrich) according to the modified method of Seskar et al. (1998).

### Volatile compound content

Solid-phase microextraction (SPME) used for determination of VOC was carried out with the method of Carlin et al. (2016). Three grams of freshly harvested leaves was incubated in 20-ml headspace vials at 40 °C for 30 min. Subsequently, extraction was performed using 50/30 divinylbenzene/carboxen/polydimethylsilox (DVB/CAR/PDMS) 1-cm long fiber for 60 min and after that the samples were introduced into gas chromatograph injection port and desorbed at 240 °C with a split ratio 1:30. All samples were analyzed by GCxGC TOF-MS. Analysis was performed using Pegasus 4D mass spectrometer equipped with a consumable-free dual-stage, quad-jet thermal modulator (LECO Corp.). A BPX5 (30 m, 0.25 mm, 0.25 μm) was used as a first-dimension (1D) column, and a BPX50 (2 m, 0.1 mm, 0.1 μm) was used as a second-dimension (2D) column. The carrier gas was helium, constant flow 1 ml/min. Temperature program conditions were as follows: first oven +50 (3 min)–280 °C at 4 °C/min, second oven, and modulator, respectively, +5 and +20 °C relatively to the first oven program (modulation time 8 s, hot pulse time 2.4 s, cold pulse −80 °C, time 1.6 s). TOF mass spectrometer parameters included mass range of m/z 33–550 at 30 spectra/s, ionization energy 70 eV, and ion source temperature 200 °C. The relative VOC content was estimated on the basis of peak areas obtained for these compounds in the control, Rs, TRS25, and TRS25 + Rs plants.

### qRT-PCR analysis of defense genes of pathogenesis-related proteins

To evaluate expression of defense-related genes of PR proteins, 200 mg of fresh cucumber leaves was frozen in liquid nitrogen and grind immediately. RNA was extracted individually from the samples of six replicates for each treatment. Total RNA was extracted with RNAzol® RT (Sigma-Aldrich) according to the instruction of the manufacturer with modifications. To precipitate RNA, supernatant was mixed with one volume of the mixture of isopropanol and NaCl-citrate buffer (0.2-M NaCl, 0.02-M sodium citrate) (1:1; *w*:*w*) to remove sugars, phenolics, and pollutants. The denaturing electrophoresis according to Masek et al. (2005) was performed to confirm the integrity of RNA structure. Extracted RNA was treated with DNase (Ambion). Subsequently, DNA-free total RNA was converted into cDNA using TranScriba Kit (A&A Biotechnology) with oligo-dT primers according to the manufacturer’s instructions.

The primers used in qRT-PCR analysis are presented in Table 1. PCR reactions were carried out in 200-μl eppendorfs. The reaction mixture contained 3 μl of diluted cDNA, 9 μl of real-time 2xPCR Master Mix SYBR® A (A&A Biotechnology), 4-μl primers mix (oligo.pl), and 4-μl DEPC water. Efficiency of primers was calculated by the dilution method. Quantitative real-time PCR was performed using the Rotor-Gene® Q Detection System at 95 °C for 5 min followed by 40 cycles at 95 °C for 15 s and at 60 °C for 1 min. UBI-ep (Ubiquitin extension protein) and TUA (α-Tubulin) genes were chosen as the most stable reference genes in the used analytical system. Relative expression of genes of interest was calculated according to Pfaffl (2001).

**Table 1:** Gene-specific primer pairs used in the qRT-PCR experiment

Gene	Forward primer	Reverse primer	Reference
*PR 4*	5′-TGGTCACTGCAACCCTGACA-3′	5′-AGTGGCCTGGAATCCGACT-3′	Alizadeh et al. 2013
*PR 12*	5′-AATGGATCCATGGCTAAGTTGCTTCCATC-3′	5′-AATGAATTCAATACACACGATTTAGCACC-3′	Hwangbo et al. 2016
*PR 1*	5′-TGCTCAACAATATGCGAACC-3′	5′-TCATCCACCCACAACTGAAC-3′	Alizadeh et al. 2013
*PR 5*	5′-CATTCTGCCTTTGTGCTTTTTC-3′	5′-ATTGATCGTCACGGTCTCGCC-3′	Liu et al. 2015
Reference gene			
*UBI-ep*	5′-CACCAAGCCCAAGAAGATC-3′	5′-TAAACCTAATCACCACCAGC-3′	Wan et al. 2010
*TUA*	5′-ACGCTGTTGGTGGTGGTAC-3′	5′-GAGAGGGGTAAACAGTGAATC-3′	Wan et al. 2010

### Callose and lignin deposition and location

To determine callose content, 500 mg of cucumber fresh leaves was homogenized with 95% EtOH (1:5; *w*:*v*) and centrifuged (15,000 ×*g*, 20 min). The supernatant was discarded and the pellet was dissolved in 0.4 ml of 1-M NaOH and incubated at 80 °C for 30 min to solubilize the callose. After centrifugation at room temperature (12,000 ×*g*, 20 min), the supernatant was used for callose determination which was performed according to Kőhle et al. (1985). The reaction mixture contained 0.4 ml of the supernatant, 0.8-ml 0.1% aniline blue, 0.42-ml 1-M HCl, and 1.18-ml 1.0-M glycine-NaOH. After incubation at 50 °C for 30 min and subsequently at room temperature for 30 min under shaking conditions, fluorescence was quantified at an excitation wavelength of 400 nm and an emission wavelength of 485 nm. Amounts of callose were expressed as *Euglena gracilis* β-1,3-glucan (Sigma-Aldrich) equivalent in mg per g of FW. For histochemical detection of callose deposition, plant tissue fragments were washed with 25-mM phosphate buffer (pH 8.2) and stained with 0.1% aniline blue prepared with 100-mM phosphate buffered saline (PBS) (pH 8.2) for approximately 20 min according to Kaźmierczak (2008). The tissue fragments were then immediately viewed in a fluorescent confocal microscope using a UV filter and excitation and emission wavelengths of 480 and 570 nm, respectively.

Lignin compounds were extracted according to the method given by Bruce and West (1989) and Mandal (2010) with modifications. Five hundred milligrams of cucumber tissue was homogenized in 80% MeOH (1:10; *w*:*v*) and centrifuged (15,000 ×*g*, 20 min). The pellet was washed three times with 80% MeOH and dried at 60 °C for 24 h. Fifteen milligrams of the insoluble residue was mixed with 5 ml of 2-M HCl and 0.5 ml of TGA, boiled in water bath for 4 h, and centrifuged (20 min, 10,000 ×*g*). The supernatant was drained out. The pellet was washed two times in distilled water, then suspended in 2.5 ml of 0.5-M NaOH, and allowed to shake on an orbital shaker for 2 h at 25 °C followed by centrifugation (20 min, 10,000 ×*g*). The supernatant was then mixed with 1 ml of concentrated HCl and thioglycolic acid (TGA) (90:1; *v*:*v*) and allowed to precipitate at 4 °C for 4 h. An orange brown pellet obtained after discarding the supernatant was dissolved in 0.5-N NaOH. After absorbance measuring at 280 nm, the quantification was based on the calibration curve for lignin standard (alkali, 2-hydroxy-propyl ether) (Sigma-Aldrich) and the content was expressed as μg per g of FW. Histochemical detection of lignin deposition was prepared according to Drnovšek et al. (2005). Plant tissue fragments were washed with 80% EtOH and stained with 1% acridine orange prepared with 96% EtOH for approximately 30 min. Then, the samples were washed in 25% HCl*.* The tissue fragments were then immediately viewed in a fluorescent confocal microscope using a UV filter and excitation and emission wavelengths of 488 and 540 nm, respectively.

### Statistical analyses

To determine cucumber shoot FW and root DW as well as to analyze biochemical parameters, the results from four independent, not significantly different trials, were combined. In all experiments, six replicates for each variant were obtained. Statistical analysis of variance (ANOVA, *P* < 0.05) for each parameter was followed by the Duncan multiple range post hoc test. Respective significant differences were marked using letters a, b, c, and d. The analyses were performed in Statistica, Version 10: New Features and Enhancements.

## Results

### Impact of *Trichoderma* on *R. solani* infection and cucumber growth

**Fig. 1:**
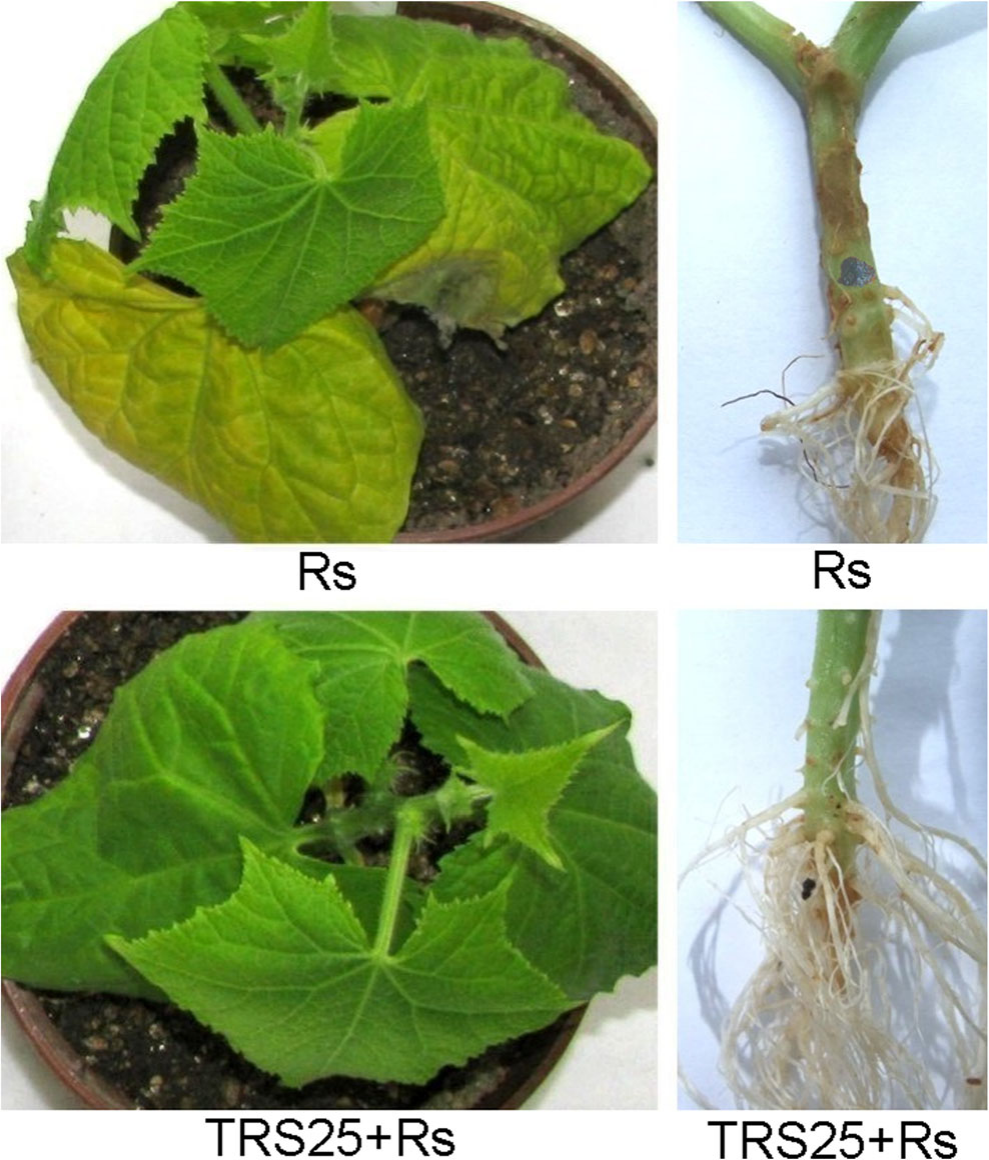
Suppression of *R. solani*-induced disease spreading on cucumber roots, shoots, and leaves by TRS25. Irregular brown lesions and rot symptoms on the roots of Rs plants were followed by shoot and leaf dark brown blight blotches and plant collapse. Abbreviations: Rs plants, TRS25 nontreated, inoculated with *R. solani*; TRS25 + Rs plants, TRS25 pretreated, inoculated with *R. solani*

**Fig. 2:**
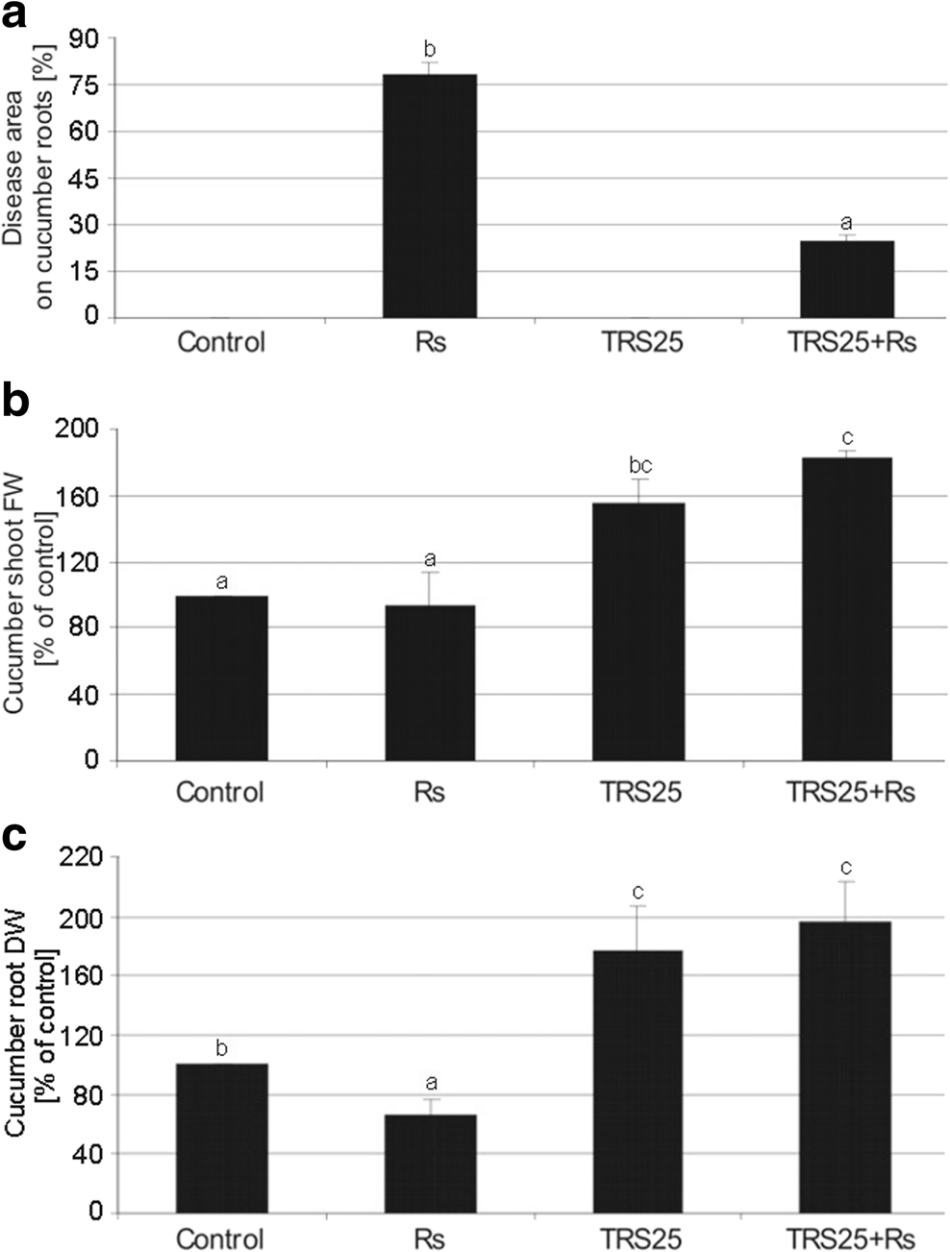
Rating of disease caused by *R. solani* (**a**) and effect of *R. solani* and TRS25 treatments on cucumber shoot fresh weight (FW) (**b**) and root dry weight (DW) (**c**). Values represent the means + SE from three independent experiments with six replicates each. Statistical analysis of variance (ANOVA, *P* < 0.05) for each parameter was followed by the Duncan multiple range post hoc test. Respective significant differences were marked using letters a, b, c, and d. Control plants (TRS25 nontreated, uninoculated with *R. solani*), Rs plants (TRS25 nontreated, inoculated with *R. solani*), TRS25 plants (TRS25 pretreated, uninoculated with *R. solani*), and TRS25 + Rs plants (TRS25 pretreated, inoculated with *R. solani*) were used in the study

*R. solani* effectively infected cucumber plants nontreated with *Trichoderma* (Rs plants). Irregular brown lesions and rot symptoms on the roots of these plants were followed by shoot and leaf dark brown blight blotches and plant collapse (Fig. 1). The development of disease symptoms in the roots of Rs plants oscillated approximately 75% above the uninfected control (Fig. 2a). Rs plants had underdeveloped and dense root system, which established average 40% lower DW in comparison to the control plants (Fig. 2c). The significant disease suppression down to mean 18% of the root area was observed in the plants pretreated with *T. atroviride* TRS25 and inoculated with *R. solani* (TRS25 + Rs plants) as compared to Rs plants (Fig. 2a). Simultaneously, TRS25 suppressed negative effect of *R. solani* on cucumber growth and plant collapse (Fig. 1). Both TRS25 and TRS25 + Rs plants had better developed root system and above ground parts. TRS25 caused pronounced increase in FW of the shoots on average by 55 and 82% (Fig. 2b) and increase in DW of the roots on average by 77 and 96% (Fig. 2c) in TRS25 and TRS25 + Rs plants as compared to the control and Rs plants, respectively.

### Biochemical, molecular, and structural hallmarks of TISR against *R. solani* induced in cucumber by TRS25

**Fig. 3:**
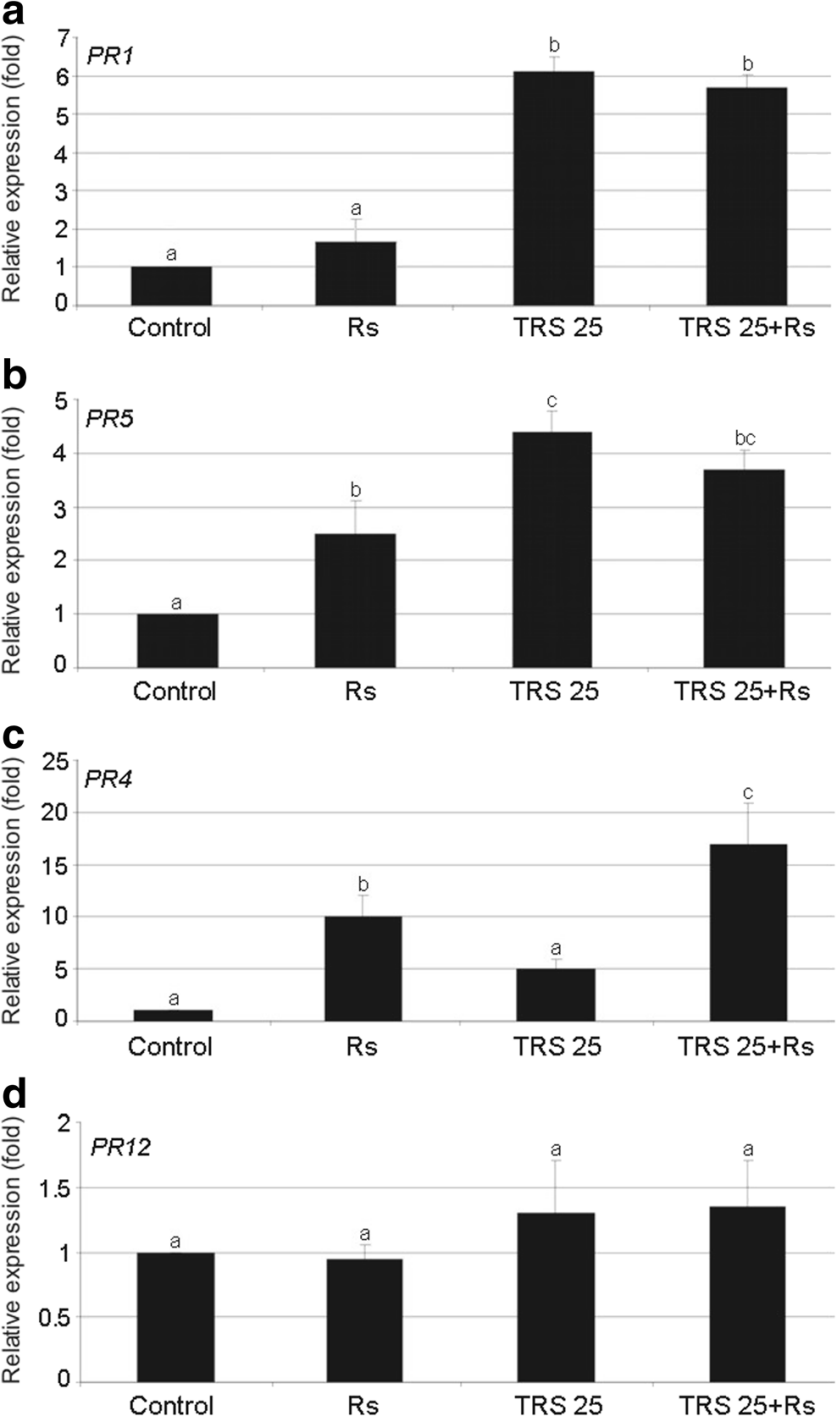
Effect of TRS25 on relative expression of defense genes: **a** *PR1*, **b** *PR5*, **c** *PR4*, and **d** *PR12* in cucumber plants. Values represent the means + SE from three independent experiments with six replicates each. Statistical analysis of variance (ANOVA, *P* < 0.05) for each parameter was followed by the Duncan multiple range post hoc test. Respective significant differences were marked using letters a, b, c, and d. Control plants (TRS25 nontreated, uninoculated with *R. solani*), Rs plants (TRS25 nontreated, inoculated with *R. solani*), TRS25 plants (TRS25 pretreated, uninoculated with *R. solani*), and TRS25 + Rs plants (TRS25 pretreated, inoculated with *R. solani*) were used in the study

*R. solani* inoculation significantly enhanced TBARS concentration (Table 2a) and decreased APX activity in Rs plants as compared to the control (Table 2b). Treatment of cucumber with TRS25 increased H_2_O_2_ content and decreased that of TBARS in TRS25 and TRS25 + Rs plants as compared to the control and Rs plants (Table 2a). Simultaneously, in the same plants, TRS25 significantly decreased APX activity and increased PAL and PPO activities, while no significant differences were detected for these parameters between TRS25 and TRS25 + Rs variants. Moreover, in TRS25 + Rs plants, additional enhancement of GPX and SPX activities was observed as compared to the control, Rs, and TRS25 plants (Table 2b). Along with induction of PAL and PPO activities, accumulation of PC in the form of *o*DP and PP both in TRS25 and TRS25 + Rs plants and accompanied with the increase in FL in the TRS25 + Rs group as compared to the control and Rs plants was detected (Table 2c). The strongest, above twofold, increase was observed for PP content in TRS25 + Rs plants as compared to Rs plants. In the PP group, SAGC concentration increased both in TRS25 and TRS25 + Rs plants as compared to the control and Rs plants (Table 2c). *R. solani* inoculation induced generation of Z-3-hexanal and E-2-hexenal, two VOC compounds which were not detected in the control plants and it increased Z-3-hexenol concentration as compared to the control (Table. 2c). The significant increases in Z-3-hexanal and E-2-hexenal accompanied by accumulation of MeSA, EHS, and β-cyclocitral were detected both in TRS25 and TRS25 + Rs plants, respectively, to the control and Rs plants (Table 2c). The increase in Z-3-hexenol concentration was additionally observed in TRS25 + Rs plants as compared to the control (Table 2c). The strongest 2.5-fold increase in MeSA content was detected in TRS25 plants and the strongest increases in EHS (3.11-fold), β-cyclocitral (2.99-fold), Z-3-hexanal (2.14-fold), Z-3-hexenol (1.84-fold), and E-2-hexenal (2.21-fold) contents in TRS25 + Rs plants. The molecular analysis revealed that *R. solani* inoculation triggered increase in *PR5* and *PR4* expression in Rs plants as compared to the control (Fig. 3b and c). Among all tested genes, TRS25 significantly up-regulated expression of *PR1* and *PR5* genes in TRS25 and TRS25 + Rs plants as compared to the control and Rs plants (Fig. 3a and b). The increases of both genes in TRS25 plants did not differ from those observed in TRS25 + Rs plants. In the presence of *R. solani*, additional *PR4* gene expression increase was observed in TRS25 + Rs as compared to the control and Rs plants (Fig. 3c). In the same plants, neither TRS25 nor *R. solani* influenced *PR12* gene expression (Fig. 3d).

**Table 2:** Effect of TRS25 on (a) hydrogen peroxide (H_2_O_2_) and thiobarbituric acid reactive substance, hallmarks of lipid peroxidation (TBARS) content, (b) ascorbate, guaiacol and syringaldazine peroxidase (APX, GPX, and SPX), phenylalanine ammonia lyase (PAL) and polyphenol oxidase (PPO) activities, (c) phenolics (PC), *orto*dihydroxyphenolics (*o*DP), phenylpropanoids (PP), favonoids (FL), free salicylic acid (SA), salicylic acid glucosylated conjugates (SAGC), and volatiles: methyl salicylate (MeSA), ethylhexyl salicylate (EHS) β-cyclocitral, Z-3-hexanal, Z-3-hexenol and E-2-hexenal content, (d) callose and lignin content

Biochemical parameter	Control		Rs		TRS25		TRS25 + Rs	
a								
H_2_O_2_ (μmol g^−1^ FW)	2.20 ± 0.38	a	2.16 ± 0.31	a	3.25 ± 0.48	b	3.13 ± 0.46	b
TBARS (nmol g^−1^ FW)	16.75 ± 4.07	b	20.76 ± 2.44	c	12.43 ± 0.58	a	12.71 ± 3.32	a
b								
Defense enzymes								
APX (U mg^−1^ protein)	2.42 ± 0.55	c	0.90 ± 0.54	a	1.51 ± 0.28	b	1.50 ± 0.31	b
GPX (U mg^−1^ protein)	1.00 ± 0.11	a	1.13 ± 0.13	a	0.99 ± 0.07	a	1.52 ± 0.11	b
SPX (U mg^−1^ protein)	0.31 ± 0.07	ab	0.39 ± 0.10	b	0.23 ± 0.06	a	0.54 ± 0.04	c
PAL (U mg^−1^ protein)	0.57 ± 0.07	a	0.82 ± 0.11	ab	1.22 ± 0.21	bc	1.46 ± 0.25	c
PPO (U mg^−1^ protein)	1.84 ± 0.36	a	2.11 ± 0.17	a	4.04 ± 1.02	c	3.84 ± 0.57	bc
c								
Metabolic components								
PC (mg g^−1^ FW)	4.87 ± 1.01	a	4.37 ± 1.22	a	9.84 ± 2.11	b	9.66 ± 1.15	b
*o*DP (mg g^−1^ FW)	0.76 ± 0.09	a	0.75 ± 0.20	a	0.90 ± 0.05	b	1.11 ± 0.17	b
PP (mg g^−1^ FW)	3.22 ± 0.28	a	2.70 ± 0.55	a	5.08 ± 0.76	b	5.40 ± 1.00	b
FL (mg g^−1^ FW)	0.25 ± 0.02	a	0.27 ± 0.03	a	0.30 ± 0.03	a	0.37 ± 0.02	b
SA (μg g^−1^ FW)	2.98 ± 0.21	a	1.88 ± 0.15	a	2.95 ± 0.34	a	1.98 ± 0.07	a
SAGC (μg g^−1^ FW)	2.95 ± 0.31	a	3.12 ± 0.17	a	5.88 ± 0.41	b	6.31 ± 0.19	b
VOC								
MeSA (fold)	1.00 ± 0.00	a	1.50 ± 0.01	a	2.50 ± 0.05	c	2.00 ± 0.04	b
EHS (fold)	1.00 ± 0.00	a	1.17 ± 0.13	a	2.25 ± 0.08	b	3.11 ± 0.09	c
β-cyclocitral (fold)	1.00 ± 0.00	a	1.11 ± 0.08	a	3.11 ± 0.12	b	2.99 ± 0.14	b
Z-3-Hexanal (fold)	0.00 ± 0.00	a	1.10 ± 0.00	b	0.98 ± 0.20	b	2.14 ± 0.33	c
Z-3-Hexenol (fold)	1.00 ± 0.00	a	1.50 ± 0.21	bc	1.14 ± 0.17	ab	1.84 ± 0.36	c
E-2-Hexenal (fold)	0.00 ± 0.00	a	1.05 ± 0.00	b	1.10 ± 0.10	b	2.21 ± 0.17	c
d								
Structural barriers								
Callose (μg g^−1^ FW)	300 ± 15	ab	248 ± 20	a	415 ± 17	c	421 ± 7	c
Lignin (mg g^−1^ FW)	1.05 ± 0.12	a	1.29 ± 0.05	ab	2.25 ± 0.09	c	1.87 ± 0.08	c

Values represent the means + SE from three independent experiments with six replicates each. Statistical analysis of variance (ANOVA, *P* < 0.05) for each parameter was followed by the Duncan multiple range post hoc test. Respective significant differences were marked using letters a, b, c, and d. Control plants (TRS25 nontreated, uninoculated with *R. solani*), Rs plants (TRS25 nontreated, inoculated with *R. solani*), TRS25 plants (TRS25 pretreated, uninoculated with *R. solani*), and TRS25 + Rs plants (TRS25 pretreated, inoculated with *R. solani*) were used in the study

**Fig. 4:**
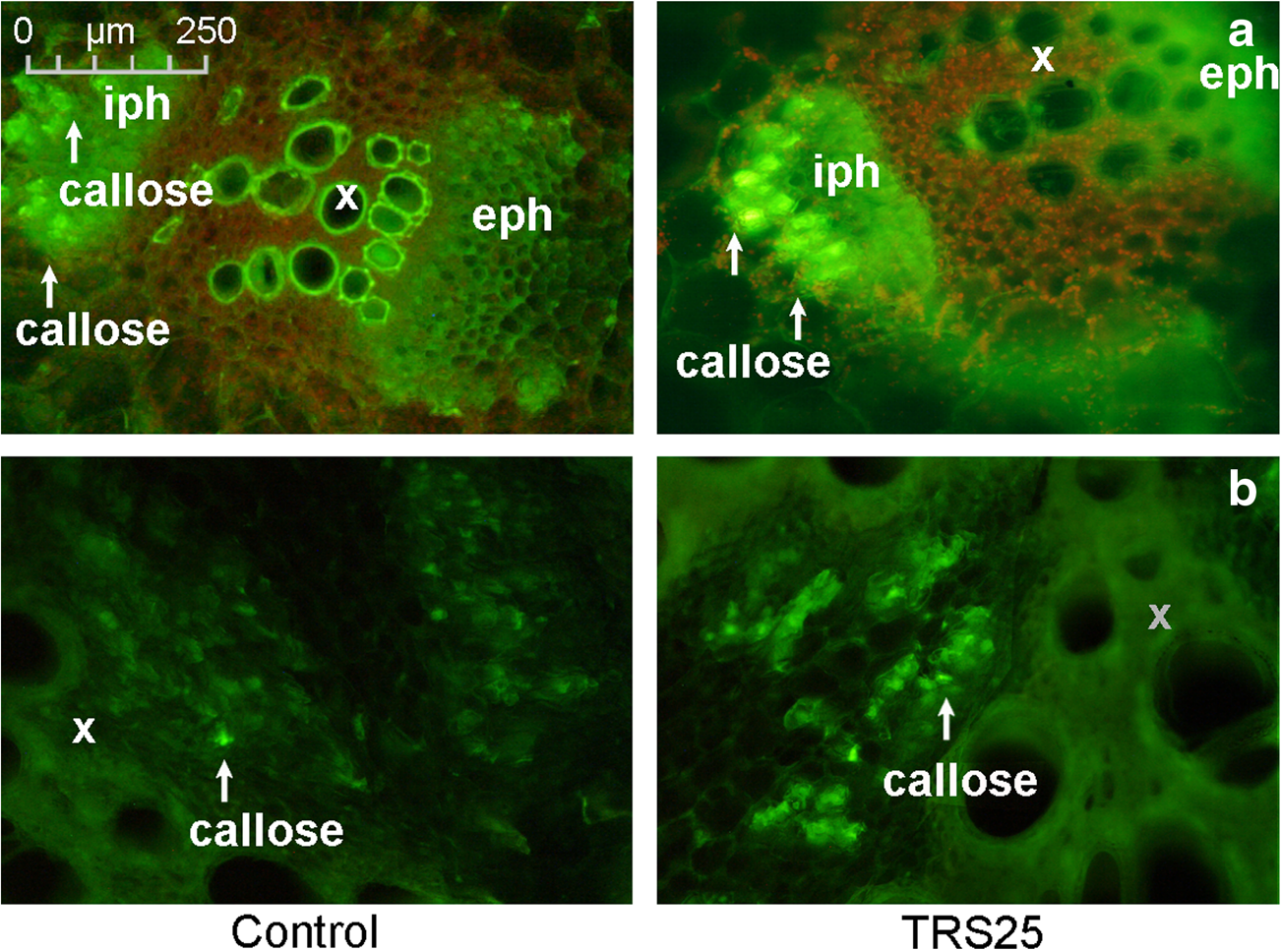
The cross section of the cucumber shoot (**a**) and root (**b**) tissues. Fluorescent confocal microscopy was used to detect callose deposition in the control and TRS25 plants. Abbreviations: iph internal phloem, eph external phloem, x xylem. The white arrows indicate light green fluorescence of callose in the cells separating internal phloem from the shoot pith in the cucumber shoot vascular bundles (**a**) and in tissue between xylem vessels of adjacent vascular bundles in roots (**b**)

**Fig. 5:**
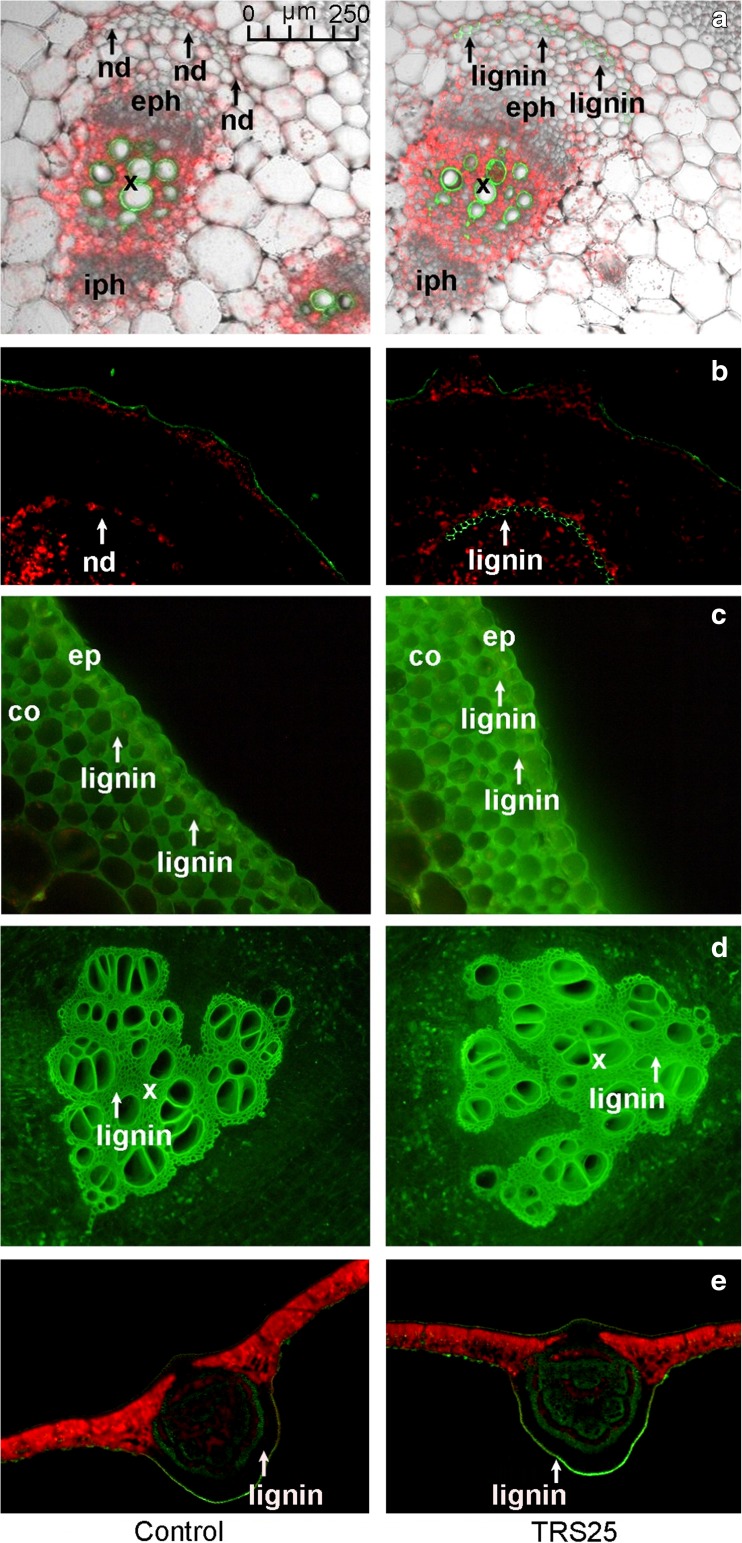
The cross section of the cucumber shoot (**a**, **b**, **c**), root (**d**), and leaf (**e**) tissues. Fluorescent confocal microscopy was used to detect lignin deposition in the control and TRS25 plants. Abbreviations: iph internal phloem, eph external phloem, x xylem, nd nondetected, ep epidermis, co collenchyma. The white arrows indicate green fluorescence of lignin detected in cells surrounding of external phloem in vascular bundles in the cucumber shoot (**a**, **b**), in the walls of epidermis and collenchyma cells in the cucumber shoot (**c**), and in epidermis of leaves (**e**)

Simultaneous to biochemical and molecular response, the present study demonstrated significant increases in concentrations of callose and lignin in TRS25 and TRS25 + Rs plants as compared to the control and Rs plants, while there were no differences in the accumulation of these compounds between TRS25 and TRS25 + Rs plants (Table 2d). The changes were not observed in Rs plants. The microscopic analyses showed that in TRS25 plants, strong fluorescence signal of callose was detected in vascular bundles in shoots where the polysaccharide was accumulated as punctate distribution patterns in the cells separating internal phloem from the shoot pith (Fig. 4a) as well as in the tissue between xylem vessels of adjacent vascular bundles in roots (Fig. 4b). Enhanced deposition of lignin appeared in shoot, root, and leave tissues of *Trichoderma*-treated plants. In these plants, the polymer was accumulated in the outer layer of external phloem in shoot vascular bundles, while it was not detectable in the control plants (Fig. 5a and b). Strong accumulation of lignin as compared to the control was also observed in the xylem vessels in roots as well as in walls of epidermis and collenchyma cells in shoots (Fig. 5c) and leaves (Fig. 5e).

## Discussion

The present study showed that *R. solani* directly infected cucumber roots. Then, the disease spread systemically in tissues, which had no contact with the pathogen leading to seedling growth inhibition and plant collapse. The observed changes were similar to root rot and foliar blight caused by *R. solani*, which were described previously in cucumber, rice, and tomato plants (Singh et al. 2002; Bartz et al. 2010; Yousef et al. 2013; Małolepsza et al. 2017). Here, we showed that application of TRS25 significantly reduced disease incidence through activation of TISR and simultaneously it promoted plant growth. The ability of the tested TRS25 strain to promote plant growth and to induce resistance may be explained by the suggestion that some *Trichoderma* strains can activate plant secondary metabolism simultaneously limiting “costs” of primary metabolism. The chemical substances released into the rhizosphere by *Trichoderma* may directly stimulate plant growth and productivity as well as enhance solubilization and uptake of microelements, nutrients, and water by plants (Harman et al. 2004; Alfano et al. 2007; Azarmi et al. 2011; López-Bucio et al. 2015). Moreover, mechanical strengthening of dermal layers and vascular system induced by TRS25 seem to be crucial for plant growth promotion, water and nutrient transport improvement and proper turgor maintaining, as well as enhancement of plant protection against *R. solani*-induced disease.

The protection of plants by *T. atroviride* might be triggered by multiple secondary metabolites released by the fungus, which influence both pathogens and plants (Contreras-Cornejo et al. 2009; Tucci et al. 2011; López-Bucio et al. 2015). The preliminary study showed that despite the fact that TRS25 released different volatiles, it had moderate ability to directly kill or antagonize *R. solani*. It did not reduce growth and did not overgrow mycelium of this pathogen (Nawrocka et al. 2011; Szczech et al. 2014)*.* In the present study, TRS25 suppressed the expression of the disease in cucumber roots where, to a certain extent, it might impede *R. solani* proliferation. However, suppression of systemic lesions, blight blotches, and rot symptoms in tissues which were not infected by the pathogen seemed to be related to TISR as in the case of cucumber, tomato, and *Arabidopsis thaliana* protected, respectively, against *Pseudomonas syringae*, *Fusarium oxysporum*, and *Botrytis cinerea* by different *Trichoderma* strains (Yedidia et al. 2003; Ozbay et al. 2004; Mathys et al. 2012). Enhancement of defense enzyme activities, accumulation of metabolic components including PC and VOC accompanied by stimulation of defense gene expression, and structural barrier strengthening were studied as symptoms of multilayer TISR. It was showed that TRS25 protected cucumber cells against membrane destruction and increased H_2_O_2_ up to the beneficial level that allowed it to act as a resistance signaling molecule, activator of protective genes, and as a substrate for defense enzymes (Gayoso et al. 2010; Djébali et al. 2011; Harman et al. 2012; Singh et al. 2011). Simultaneously, as in previous reports of Singh et al. (2011), Solanki et al. (2011), and Surekha et al. (2013) focusing on different *Trichoderma* strains, in the present study lipid peroxidation decrease might result from additional protection of cells by GPX and SPX strongly involved in plant cell wall strengthening during, e.g., lignification as well as from increase in PC synthesis as a result of enhanced PAL and PPO enzyme activities induced by TRS25. The important role of overproduced *o*DP, PP, and FL in TISR and direct suppression of pathogens as a result of their antioxidative or antibiotic properties were presented previously (Shoresh et al. 2005; Lattanzio et al. 2006; Harman et al. 2012; Nikraftar et al. 2013; Zhang et al. 2016). Moreover, as described by Gayoso et al. (2010), Solanki et al. (2011), and Nikraftar et al. (2013), PC interacting with PR proteins and defense enzymes might participate in toughening of cell walls during lignification process.

In the recent years, it has been documented that TISR induced against different pathogens may involve a complex network of cross-communicating signaling molecules. Most of the studies focused on the relationship between jasmonic acid (JA), ethylene (ET), and SA and their derivatives (Yedidia et al. 2003; Shoresh et al. 2005; Segarra et al. 2007; Salas-Marina et al. 2011; Tucci et al. 2011; Martinez-Medina et al. 2013; Perazzolli et al. 2012). The preliminary study showed no changes in the content of precursors of JA including octadecanoid and octadecatrienoic acids as well as no significant synthesis of methyl jasmonate (MeJA) derivative and of ET in TRS25 and TRS25 + Rs plants (data not shown). On the other hand, the strain strongly stimulated accumulation of hybroxybenzoic acids being precursors of SA (Nawrocka et al. 2017). In the present study, we assessed involvement of different forms of PC including SA as well as VOC newly identified in *Trichoderma* pretreated plants in resistance induction against *R. solani*. Among SA derivatives, TRS25 significantly enhanced accumulation of MeSA, which is one of the molecules involved in resistance signaling characteristic to SAR (Park et al. 2007; Vogt 2010; Gao et al. 2014). Moreover, the excess of SA seemed to be accumulated as SAGC, which is considered as a storage form of this compound releasing SA when necessary (Takatsuji et al. 2014) or volatile EHS, one of natural pesticides rarely detected in plants (Yedida et al. 2003; Vogt 2010 Singh et al. 2011; Oliveira et al. 2016). The increase in the concentration of different SA derivatives in TRS25 plants was accompanied by significant accumulation of another VOC, β-cyclocitral, which recently was found to up-regulate SA signaling (Lv et al. 2015). The accumulation of all SA derivatives and VOC, mentioned above, was also observed in TRS25 + Rs plants with significantly reduced *R. solani*-induced disease symptoms. Moreover, in these plants, simultaneous generation of unsaturated fatty acid derivatives including Z-3-hexanal, Z-3-hexenol, and E-2-hexenal was detected. In correlation with other VOC including SA derivatives, these compounds might be among the fastest signaling molecules able to directly elicit or prime plant systemic defense response and resistance. According to Scala et al. (2013), VOC signals seem to be faster than vascular signal molecules and can also reach organs not directly connected through vascular system. Thus, in this model, vascular and airborne signals may act synergistically to ensure optimal resistance in distal plant parts. Moreover, the present VOC, especially E-2-hexenal and Z-3-hexenol with antimicrobial activity, might prevent microbes from invading plants as well as participate in stronger lignification of plant tissues (Scala et al. 2013). All the mentioned VOC together with H_2_O_2_ may be involved in resistance signaling resulting in enhanced expression of PR proteins (Mathys et al. 2012; Brotman et al. 2013; Scala et al. 2013; Gao et al. 2014; Lv et al. 2015; De Palma et al. 2016; Adrian et al. 2017). Since the genes encoding PR proteins may be up-regulated by different signaling molecules, their expression is considered as a marker of the type of induced resistance (Yedidia et al. 2000; Shoresh et al. 2005; Vinale et al. 2008). In the present study, the protection of cucumber against *R. solani* was effective in the plants in which up-regulation of SAR-related *PR1* and *PR5* genes was accompanied by increase in the expression of ISR gene *PR4* in TRS25 + Rs plants, while this gene expression did not increase in TRS25 plants. On the other hand, up-regulation of *PR4*, unaccompanied by over-expression of other tested genes in Rs plants neither decreased plant susceptibility to *R. solani* attack nor induced even partial plant resistance. The complex network of resistance inducers including SA derivatives and unsaturated fatty acid derivatives present in TRS25 + Rs plants may explain the enhanced expression of genes characteristic of two kinds of systemic resistance, i.e., ISR/SAR. It is suggested that some of the genes induced by unsaturated fatty acid derivatives including E-2-hexenal and Z-3-hexenal are also induced by JA in *Arabidopsis* (Scala et al. 2013). Thus, one could suggest that MeSA, EHS, and β-cyclocitral participated in the up-regulation of *PR1* and *PR5* genes and SA-dependent response while Z-3-hexanal, Z-3-hexenol, and E-2-hexenal promoted the up-regulation of *PR4* and JA-dependent responses that help cucumber plants to counteract *R. solani*. Such stimulation was reported for Z-3-hexenal and Z-3-hexenol which induced expression of several defense JA-related genes in *Arabidopsis* such as defensin 1.2 (PDF1.2) and PR-3 (chitinase B) protecting the plant against *B. cinerea* (Kishimoto et al. 2006). Moreover, it is possible that the mentioned compounds may be accompanied by other signaling VOC from the groups of unsaturated fatty acid derivatives and PC; however, additional studies are necessary to determine them. At the moment, the observed combined type of gene up-regulation might be similar to the cross-TISR characterized by Perazzolli et al. (2012) and Levy et al. (2015) in *Trichoderma* *harzianum*-related protection of plants against *Plasmopara viticola*, *B. cinerea*, and *Podosphaera xanthii* which included SAR- and ISR-related gene expression including *PR1*, *PR2*, and *PR4* up-regulated in the presence of pathogens. The studied genes encode proteins, which may participate in plant defense response, cell wall reinforcement, and direct protection against *R. solani* as it was presented previously for plants interacting with *Trichoderma hamatum* and *T. harzianum* (Alfano et al. 2007; Tucci et al. 2011; Mathys et al. 2012).

When plants are exposed to pathogens, simultaneously to biochemical and molecular response, the mechanical strengthening of plant tissues may contribute to pathogen spread restriction (Chowdhury et al. 2014; Rao et al. 2015). Plant defense response and resistance against fungal pathogens including *R. solani* was found to partly result from stimulation of cell wall fortification by formation of lignin and callose in plants (Yedidia et al. 2000; Solanki et al. 2011; Martínez-Cortés et al. 2012; Nikraftar et al. 2013). Simultaneous involvement of peroxidase, chitinase, and lignin formation in protection of tomato and rice against *R. solani* was described by Taheri and Tarighi (2012). Moreover, the accumulation of newly formed callose beyond the sites of fungal penetration in the epidermal and cortical cell walls in the roots and leaves of cucumber plants treated with *T. harzianum* was found previously by Yedidia et al. (2000). In the present study, the contents of lignin and callose were strongly enhanced in the plants pretreated with TRS25 both in the absence and presence of *R. solani*. These components were detected in the cucumber tissues, which had no direct contact with TRS25 and *R. solani*. Since in the previous studies enhancement of structural barriers was mainly analyzed and described in the area of fungal influence (Chowdhury et al. 2014; Rao et al. 2015; Schneider et al. 2016), we focused on systemic location of callose and lignin in the whole plant. The microscopic analyses showed that main localizations of these compounds were observed in the area of vascular bundles and dermal tissues in cucumber shoot, root, and leaves where they were localized in different cells. In the cucumber shoot, callose deposited as punctate distribution patterns seemed to be integral to specialized, separated cells which protected vessels at the side of internal phloem. Lignin, which was most likely produced by SPX from accumulated PP units, was distributed regularly in the line of cell walls of an outer layer of external phloem in the vascular bundles. The lignin layer was not detectable in the control plants. The two compounds seemed to act together also in the vascular bundles of roots where lignin formed the xylem vessels, and callose was localized in separate cells between adjacent vascular bundles. Together, the compounds formed mechanical layer protecting phloem and xylem from both sides not restricting growth of other cells. They also protected the walls of epidermis and collenchyma cells in shoots and leaves from the outside environment and they made organs more flexible and resilient, helped them maintain the correct position, and protected deeper layers of assimilation cells. The obtained results suggest that callose and lignin depositions might not only be involved in protection of cucumber plants against spread of *R. solani*-induced disease and against plant cell degradation by enzymes and toxins released by the pathogen as suggested previously (Djonović et al. 2007; Huang et al. 2010; Spoel and Dong 2012; Doorn and Ketsa 2014), but simultaneously, they might play important role in plant growth promotion by reinforcement of vascular system making water and nutrient transport more effective.

## Conclusions

Taken together, the study showed that *T. atroviride* TRS25 strain was effective in growth promotion and in reduction of *R. solani*-induced disease in cucumber plants. The systemic disease suppression was not related to direct inhibition of *R. solani* growth and proliferation by TRS25 but to stimulation of multilayer TISR. Treatment of the cucumber roots with TRS25 induced biochemical, molecular, and structural changes, which are important in plant defense responses and resistance. In TRS25-induced TISR, systemic enhancement of defense enzymes including GPX, SPX, PAL, and PPO accompanied by accumulation of H_2_O_2_ and PC as well as decrease in lipid peroxidation was detected. To the best of our knowledge, the important role of accumulation of VOCs including EHS and β-cyclocitral and of unsaturated fatty acid derivatives, that is, Z-3-hexanal, Z-3-hexenol, and E-2-hexenal as well as SA storage as SAGC was observed for the first time in plants protected by *Trichoderma* against *R. solani*. We put forward a hypothesis that together with MeSA, all these compounds may enhance expression of SAR- and ISR-related genes leading to induction of mixed type of TISR. Moreover, TRS25 inducing system of callose and lignin deposition promoted mechanical plant strengthening. The in-depth microscopic analysis revealed that the detected compounds protected vascular system both at internal and external phloem sides in cucumber shoots. Lignin formed the wall thickening xylem vessels, and callose was localized in separate cells between adjacent vascular bundles in roots. These compounds protected cells and dermal tissues in cucumber roots, shoots, and leaves against *R. solani* and made them more flexible and resilient, which helped to maintain turgor and contributed to better nutrition and hydration of plants. The growth promotion coupled with systemic mobilization of biochemical, molecular, and structural response might be positively involved in multilayer protection of cucumber plants against *R. solani* activated by TRS25. Based on the results, TRS25 strain has potential to be an effective biocontrol agent for *R. solani*-induced disease management in cucumber plants.
